# You can teach an old dog new tricks: angiopoietin-1 instructs Tie2^pos^ myeloid cells to promote neovascularization in ischemic limbs

**DOI:** 10.1002/emmm.201302794

**Published:** 2013-06-04

**Authors:** Costanza Emanueli, Nicolle Kränkel

**Affiliations:** 1 Laboratory of Vascular Pathology and Regeneration, School of Clinical Sciences, University of BristolBristol, United Kingdom; 2 Vascular Science, National Heart & Lung Institute, Imperial College of LondonLondon, United Kingdom; 3 University of Zurich, Institute of Physiology, Working Group on Cardiovascular ResearchZurich, Switzerland; 4 Department of Cardiology, University Hospital ZurichZurich, Switzerland

**Keywords:** limb ischemia, therapeutic neovascularization, angiopoietin-1, PHD2, myeloid cells

See related articles in EMBO Molecular Medicine http://dx.doi.org/10.1002/emmm.201302695 and http://dx.doi.org/10.1002/emmm.201302752

Throughout life, the vasculature undergoes remodelling to adapt to the changing demands. The ability to grow new capillaries, adapt their caliber to higher flow and develop collaterals from obstructed arteries is essential to maintain or restore tissue perfusion. Better knowledge of the molecular and cellular details of postnatal blood vessel growth and remodelling is necessary to develop improved vascular regenerative therapies.

Despite the crucial roles of myeloid cells in immune defense and angiogenesis, we still lack a thorough understanding of their identity and functional regulation.

In this issue of EMBO Molecular Medicine, two complementary articles reinforce the notion that Angiopoietin-1 (Ang-1) and Tie2 receptor expressing monocytes/macrophages (TEM) team up to promote reparative vascularization in limb ischemia (LI; Hamm et al, 2013; Patel et al, 2013). The underpinning molecular mechanism is an inhibitory effect of Ang-1 (a natural ligand for Tie2) on the hypoxia inducible factor-1 α (HIF-1α) destabilizer factor prolyl hydroxylase 2 (PHD2) in TEM (Hamm et al, 2013; Fig. 1). It was already known that Ang-1 promotes angiogenesis in ischemic limbs (Shyu et al, 1998). Moreover, *ex vivo* priming with Ang-1 improves the vasculogenic potential of peripheral blood (PB) stem cells and increases their engraftment and therapeutic angiogenesis response after transplantation in a mouse model of LI (Kim et al, 2009).

**Figure 1:**
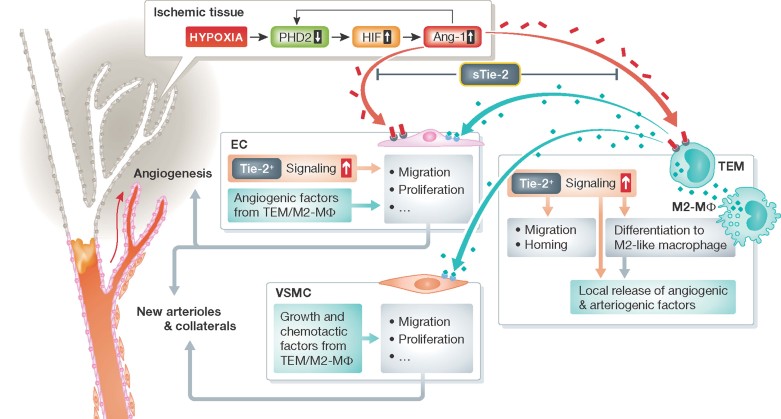
Molecular and cellular mechanisms initiating by Angiotensin-1 and leading to post-ischemic vascular repair In ischemic tissues, the activity of the oxygen sensor PDH2 is reduced, permitting the HIFα-mediated transcription of pro-angiogenic genes, such as Ang-1 (red). In a feedback loop, Ang-1 after binding with its Tie2 receptor, represses PHD2. This leads to increased circulating and ischemic tissue-engrafted TEM and skews macrophages to the proangiogenic M2 type. TEM and M2 release pro-angiogenic/pro-arteriogenic factors (blue) acting on endothelial cells (EC) and vascular smooth muscle cells (VSMC) to promote cellular processes leading to formation of capillaries, arterioles and arterial collaterals. Additionally, Ang-1 and Ang-2 binding to Tie2 receptor regulates angiogenesis at EC level. Soluble Tie2 (sTie2) inhibits Ang/Tie2 signalling in ischemia, thus contrasting vascular repair.

» Better knowledge of the molecular and cellular details of postnatal blood vessel growth and remodelling is necessary to develop improved vascular regenerative therapies. «

TEM were known to infiltrate tumours and promote cancer angiogenesis in a paracrine manner (De Palma et al, 2005). These new studies (Hamm et al, 2013; Patel et al, 2013) suggest the possibility that TEM are utilized by both tumours and ischemic tissues to increase their incoming blood flow. TEMs differ from Tie-2^neg^ tumour-associated myeloid cells by their pro-angiogenic function and gene expression signature, which resemble that of ‘alternatively activated/regenerative’ M2-like macrophages, rather than the ‘classically activated/inflammatory’ M1 macrophages (Pucci et al, 2009). However, due to the high plasticity of myeloid cells – including fast internalization or upregulation of surface receptors, *e.g.* during sample processing – direct conclusions from antigen makeup of the macrophage to its function should be made with caution.

PHD2 is an oxygen sensor and supports the degradation of α subunits of HIF complexes and hence inhibits the expression of HIF-1α -target genes, including Ang-1. *Phd2*^−/−^ mice show diffuse hyperactive angiogenesis, angiectasia and enhanced recruitment of vascular smooth muscle cells (VSMCs) in subendocardial medium–sized vessels (Takeda et al, 2007). Moreover, PHD2 haplodeficiency (*Phd2*^+/−^) skews macrophages to M2 type, increases M2 macrophage engraftment and their release of arteriogenic factors in limb muscles, thus leading to enhanced VSMCs recruitment and growth and arterial collateralization under non-ischemic conditions in mice. Consequently, *Phd2*^+/−^ mice are ‘preconditioned’ to better respond once LI is experimentally induced (Takeda et al, 2011).

Hamm et al (2013) elegantly show that Ang-1, via Tie2, down-regulates PHD2 expression. Moreover, they confirm the important role of TEM in post-LI vascular repair by using a ‘cell suicide’ system. To complement this set of information, the translational study of Patel et al has characterized TEMs in the PB of human patients with critical limb ischemia (CLI), compared to age-matched controls, younger healthy individuals and patients with intermittent claudication. TEM were robustly increased in the circulation of CLI patients and this was corrected after revascularization or limb amputation (Patel et al, 2013), suggesting that TEM (or their progenitors) are able to sense and respond to LI (Hamm et al, 2013; Patel et al, 2013). This is in line with the concept that ischemic events activate bone marrow (BM) changes, including an altered production and release of monocytic cells. Does CLI enhance the BM generation of TEMs and/or other pro-angiogenic cells or merely facilitate their egress? Are specific cell types released from the BM ‘ready-made’ and successively recruited to the ischemic limb, or are their receptor expression and downstream signalling altered once the cells encounter the ischemic area, thus helping them to engraft, survive, and sustain their function? These questions need further investigations.

» TEM, Ang-1 and PHDs should be regarded as important cellular and molecular targets able to promote therapeutic neovascularization in CLI and other ischemic diseases. «

Importantly, TEM from CLI patients show an enhanced proangiogenic potential *in vitro* and in mice with LI. However, this data needs to be reconciled with the incapacity of the CLI leg to heal. In addition to the vascular occlusion, neuropathy, gangrene and infections contribute to the CLI clinical scenario. Authors speculate on a defect of TEM recruitment in CLI. However, TEM were found infiltrated in the CLI leg, where the necrotic tissue showed twice as much TEM in comparison to the ‘normal’ area, which was co-amputated during surgery (Patel et al, 2013). This suggests that local ischemia activates mechanisms promoting TEM recruitment. It is otherwise possible that the CLI environment is not conducive for TEM to induce reparative neovascularization (Patel et al, 2013). Resident vascular cells might be compromised in their response to TEM-derived factors and ‘confused’ by contradictory commands derived from different infiltrating BM-derived subpopulations and locally dying cells, thus making the interaction with TEM more feeble or distorted. Additionally, increased circulating soluble Tie2 (sTie2) might counteract TEM functions in CLI patients (Patel et al, 2013). Overall, the above suggests that TEM function and recruitment process could be improved by systemic use of synthetic PHD2 inhibitory compounds, exposure to Ang-1 and/or reducing sTie2.

In mammals, there are 3 PHD isoforms (1,2,3), which share enzymatic properties but differ by their expression profiles. PHD1 inhibition induces hypoxia tolerance in ischemic muscles by reprogramming their glucose metabolism from oxidative to more anaerobic ATP production, thus conferring protection of myofibers (Aragones et al, 2008). Moreover, local PHD3 silencing proved able to induce therapeutic neovascularization in mouse LI (Loinard et al, 2009). Therefore, inhibition of the three PHD isoforms could have synergistic therapeutic effects in CLI.

In conclusion, TEM, Ang-1 and PHDs should be regarded as important cellular and molecular targets able to promote therapeutic neovascularization in CLI and other ischemic diseases. Further investigations allowing translation to interventional clinical trials should be developed.

## References

[b1] Aragones J, Schneider M, Van Geyte K, Fraisl P, Dresselaers T, Mazzone M, Dirkx R, Zacchigna S, Lemieux H, Jeoung NH (2008). Deficiency or inhibition of oxygen sensor Phd1 induces hypoxia tolerance by reprogramming basal metabolism. Nat Genet.

[b2] De Palma M, Venneri MA, Galli R, Sergi Sergi L, Politi LS, Sampaolesi M, Naldini L (2005). Tie2 identifies a hematopoietic lineage of proangiogenic monocytes required for tumor vessel formation and a mesenchymal population of pericyte progenitors. Cancer Cell.

[b3] Hamm A, Veschini L, Takeda Y, Costa S, E D, Squadrito ML, Henze AT, Wenes M, Serneels J, Pucci F (2013). PHD2 regulates arteriogenic macrophages through TIE2 signaling. EMBO Mol Med.

[b4] Kim MS, Lee CS, Hur J, Cho HJ, Jun SI, Kim TY, Lee SW, Suh JW, Park KW, Lee HY (2009). Priming with angiopoietin-1 augments the vasculogenic potential of the peripheral blood stem cells mobilized with granulocyte colony-stimulating factor through a novel Tie2/Ets-1 pathway. Circulation.

[b5] Loinard C, Ginouvès A, Vilar J, Cochain C, Zouggari Y, Recalde A, Duriez M, Lévy BI, Pouysségur J, Berra E (2009). Inhibition of prolyl hydroxylase domain proteins promotes therapeutic revascularization. Circulation.

[b6] Patel AS, Smith A, Nucera S, Biziato D, Saha P, Attia RQ, Humphries J, Mattock K, Grover SP, Lyons OT (2013). TIE2-expressing monocytes/macrophages regulate revascularization of the ischemic limb. EMBO Mol Med.

[b7] Pucci F, Venneri MA, Biziato D, Nonis A, Moi D, Sica A, Di Serio C, Naldini L, De Palma M (2009). A distinguishing gene signature shared by tumor-infiltrating Tie2-expressing monocytes, blood ‘resident’ monocytes, and embryonic macrophages suggests common functions and developmental relationships. Blood.

[b8] Shyu KG, Manor O, Magner M, Yancopoulos GD, Isner JM (1998). Direct intramuscular injection of plasmid DNA encoding angiopoietin-1 but not angiopoietin-2 augments revascularization in the rabbit ischemic hindlimb. Circulation.

[b9] Takeda K, Cowan A, Fong GH (2007). Essential role for prolyl hydroxylase domain protein 2 in oxygen homeostasis of the adult vascular system. Circulation.

[b10] Takeda Y, Costa S, Delamarre E, Roncal C, Leite de Oliveira R, Squadrito ML, Finisguerra V, Deschoemaeker S, Bruyere F, Wenes M (2011). Macrophage skewing by Phd2 haplodeficiency prevents ischaemia by inducing arteriogenesis. Nature.

